# Morphometric and molecular characterization of populations of *Pratylenchus kumamotoensis* and *P. pseudocoffeae* (Nematoda, Pratylenchidae) newly recorded in Korea

**DOI:** 10.3897/zookeys.600.8508

**Published:** 2016-06-22

**Authors:** Dongwoo Kim, Jae-Yong Chun, Kyeong-Yeoll Lee

**Affiliations:** 1 College of Agriculture and Life Sciences, Kyungpook National University, Daegu, Republic of Korea; 2 Plant Quarantine Technology Center, Animal and Plant Quarantine Agency, Gimcheon, Republic of Korea; 3 Institute of Plant Medicine, Kyungpook National University, Daegu, Republic of Korea; 4 Sustainable Agriculture Research Center, Kyungpook National University, Gunwi, Republic of Korea

**Keywords:** Chrysanthemum cultivars, ITS, Root-lesion nematodes

## Abstract

At least 70 species of root-lesion nematodes, *Pratylenchus* spp., have been identified worldwide, many of which are serious pests of various agricultural crop plants. In Korea, only 14 species have been recorded in vegetable and fruit fields. Here, we report two new records of root-lesion nematode species in Korea based on morphometric and molecular methods. Soil samples were collected from chrysanthemum cultivars in various regions. Morphometric diagnosis showed that two new records for Korea: *Pratylenchus kumamotoensis* in Chilgok County and *Pratylenchus pseudocoffeae* in Geumsan County. In addition, molecular diagnosis using the two sequences of the internal transcribed spacer (ITS) and the D2–D3 region of ribosomal DNA showed that these two species were most similar with those from Japan, Costa Rica and USA. The similarities of the ITS and D2–D3 sequences were 99.85 and 99.74%, respectively, for *Pratylenchus kumamotoensis* and 99.99 with Costa Rica populations and 99.86% with USA populations, respectively, for *Pratylenchus pseudocoffeae*. To our knowledge, this is the first report of two species in Korea.

internal transcribed spacer

## Introduction

In 2012–2014, amphimictic root-lesion nematode populations were detected in soil and root samples from chrysanthemum (*Chrysanthemum* sp.) fields located in Chilgok and Geumsan Counties in Korea. The population from Chilgok Co. was identified as *Pratylenchus kumamotoensis* and the other from Geumsan Co. as *Pratylenchus pseudocoffeae* using morphological and molecular analyses. The phylogenetic relationship of these Korean root-lesion nematodes with other populations reported on chrysanthemum in Japan and distant geographical areas was also analyzed.

Nematodes were extracted from the soil samples using Cobb’s sieving and gravity method and the modified Baermann funnel method. Extracted nematodes in water suspension were killed by gentle heating, dehydrated by using the rapid lactophenol method and transferred in glycerin in permanent slides (Southey 1986). Nematode specimens were measured with the aid of Zeiss M1 light microscope. DNA from specimens of the two populations was extracted using the proteinase K method. Extracted DNA from a single nematode was directly used for PCR analysis.

The sequence of the ITS region, including ITS1, 5.8S, and ITS2, was amplified using the forward TW81 primer (5’-GTT TCC GTA GGT GAA CCT GC-3’) and reverse AB28 primer (5'-ATA TGC TTA AGT TCA GCG GGT-3') (Tanha Maafi et al. 2003). The sequence of the D2–D3 expansion segment of 28S rDNA was amplified using the forward D2A primer (5'-ACA AGT ACC GTG AGG GAA AGT TG-3') and reverse D3B primer (5'-TCG GAA GGA ACC AGC TAC TA-3') (Subbotin et al. 2006). The PCR products were purified using Wizard SV Gel and PCR Clean-Up System (Promega, Madison, WI, USA) and cloned using T-blunt vectors and DH5α competent cells, as described in the manufacturer’s procedure (Solgent, Daejeon, Korea). The sequences were determined using the vector internal primer sets (M13F/M13R) at the Solgent Sequencing Facility (Daejeon, Korea) with a BigDye® Terminator Cycle Sequencing Kit and ABI 3730XL DNA analyzer (Applied Biosystems, Foster City, USA). The newly obtained sequences were assembled and edited by the alignment software Clustal W version 2.0 (Larkin et al. 2007; Goujon et al. 2010; McWilliam et al. 2013) and submitted to a search for similarity in GenBank using the Blast program (Altschul et al. 1990; Morgulis et al. 2008). Multiple sequence alignment was performed using Bioedit version 7.2.5 (Hall 1999), and a phylogenetic analysis was conducted using the MEGA 6 program (Tamura et al. 2013) with the neighbor-joining method (Saitou and Nei 1987). The GenBank data of related species were also included in the phylogenetic analysis.

The morphometric characters of the Korea population of *Pratylenchus kumamotoensis* females that were measured included (n = 29) : body length = 511.3 ± 30.87 (451.9 – 564.1 μm), a = 26.7 ± 2.10 (23.2 – 31.9 μm), b = 6.9 ± 0.55 (6.0 – 7.9 μm), b’ = 5.0 ± 0.40 (4.3 – 5.8 μm), c = 20.3 ± 1.87 (17.2 – 23.9 μm), c’ = 2.4 ± 0.19 (2.0 – 2.6 μm), V(%) = 75.7 ± 1.85 (71.4 – 81.8), stylet = 14.8 ± 0.55 (13.4 – 15.7 μm).

The morphometric characters obtained for the Korean *Pratylenchus pseudocoffeae* females included (n = 36) : body length = 565.9 ± 51.99 (475.3 – 660.4 μm), a = 23.1 ± 1.67 (20.7 – 29.2 μm), b = 6.8 ± 0.46 (6.1 – 7.7 μm), b’ = 3.7 ± 0.24 (3.2 – 4.1 μm), c = 19.4 ± 1.40 (17.3 – 21.8 μm), c’ = 2.0 ± 0.23 (1.6 – 2.4 μm), V(%) = 79.6 ± 0.80 (78.2 – 81.2), stylet = 17.2 ± 0.97 (15.9 – 19.3 μm).

These morphometrics matched those reported by Mizukubo et al. (2007) for *Pratylenchus kumamotoensis* and Mizukubo (1992) for *Pratylenchus pseudocoffeae* in Japan. Korean specimens of *Pratylenchus kumamotoensis* showed the esophageal glandular lobe aberrantly overlapping dorsally the intestine like the paratypes described in Japan. Body and stylet length of Korean *Pratylenchus kumamotoensis* were 50 μm shorter and 1 μm longer, respectively, than those of the Kanoya paratype of this species. Females of Korean *Pratylenchus pseudocoffeae* had sub-hemispherical tail with smooth terminus like the paratypes from Japan. Stylet length and distance between the base of esophageal gland lobe to the anterior body end of Korean *Pratylenchus pseudocoffeae* were 1 μm longer and 6 μm shorter, respectively, than those of the Miyazaki paratype and the populations from aster reported in Florida (USA).

The ITS regions, including ITS1, 5.8S, and ITS2 of the Korean *Pratylenchus kumamotoensis* and *Pratylenchus pseudocoffeae* were 664 and 849 bp, respectively (Suppl. material 1: Fig. S1, 2). In addition, the amplified D2–D3 regions of the two species were 762 and 737 bp, respectively (Suppl. material 1: Fig. S3, 4). The comparison of nucleotide sequences of PCR products with those of the GenBank database showed that both the ITS and D2–D3 sequences of the Korean *Pratylenchus kumamotoensis* were most similar to those of *Pratylenchus kumamotoensis* of Japan with 99.85 and 99.74% similarity, respectively. The ITS sequences of the Korean *Pratylenchus pseudocoffeae* population were most similar to those of *Pratylenchus pseudocoffeae* populations from Costa Rica, with 99.99% similarity (Araya et al. 2016), whereas the D2-D3 sequences were most similar to those of the population from USA, with 99.86% similarity (Duncan et al. 1999).

The results of the phylogenetic analysis using ITS sequences of our sample (Chilgok population) and eight populations of *Pratylenchus kumamotoensis* from Kagosima, Kumamoto, and Oita Prefectures of Japan showed that they clustered in two clades, which were not geographically separated (Suppl. material 1: Fig. S5). Namely, one clade included Kagosima, Kumamoto, and Oita samples, but the other included Kumamoto and Oita samples. Our Chilgok sample (KT175515) was least different from the Oita sample (LC030318), with a 0.15% difference, but highly different from the Kagosima sample (LC030312) with a 3.04% difference. In addition, the D2–D3 sequence of our Chilgok sample (KT175528) had a 0.26% difference with one listed sequence (JX144360) from Kumamoto (Suppl. material 1: Fig. S6). The results of the phylogenetic analysis using ITS sequences of our Geumsan sample (KT175523) and six populations of *Pratylenchus pseudocoffeae* from Costa Rica, Iran and Japan (Araya et al. 2016; De Luca et al. 2011) showed that they differed by no more than 0.35%. Our Geumsan sample was 0.12% different from the Japan sample (LC030337) and 0.35% different from the Iran sample (FR692276) (Suppl. material 1: Fig. S5). Further, it was only 1 bp different from Costa Rica sample (KT971367). In addition, the D2–D3 sequence of our Geumsan sample (KT175531) differed by 0.14% from the USA sequence (AF170444) and 0.27% from the Costa Rica sequence (KT971360) (Suppl. material 1: Fig. S6). All of the new sequences for the Korean populations of *Pratylenchus kumamotoensis* and *Pratylenchus pseudocoffeae* were deposited in GenBank with the accession numbers listed in parenthesis in the text.

To our knowledge, this is the first record of occurrence of *Pratylenchus kumamotoensis* and *Pratylenchus pseudocoffeae* in chrysanthemum fields in Korea.

## Supplementary materials

Supplementary material 1 Analysis of ITS2 and D2-d3 segment sequences of *Pratylenchus kumamotoensis* and *Pratylenchus pseudocoffeae*. Data type: Nucleotide sequences and cladograms Explanation note: Figure S1. Alignment of the ITS region of *Pratylenchus kumamotoensis*. Figure S2. Alignment of the ITS region of *Pratylenchus pseudocoffeae*. Figure S3. Alignment of the D2-D3 expansion segment of *Pratylenchus kumamotoensis*. Figure S4. Alignment of the D2-D3 expansion segment of *Pratylenchus pseudocoffeae*. Figure S5. Neighbor-joining tree for *Pratylenchus* species based on ITS sequences. Numbers adjacent to branches denote the bootstrap values (>50%) of 1000 replicates. Sequences identified in this study were indicated in bold. Figure S6. Neighbor-joining tree for *Pratylenchus* species based on D2-D3 sequences. Number adjacent to branches denote the bootstrap values (>50%) of 1000 replicates. Sequences identified in this study are indicated in bold. This dataset is made available under the Open Database License (http://opendatacommons.org/licenses/odbl/1.0/). The Open Database License (ODbL) is a license agreement intended to allow users to freely share, modify, and use this Dataset while maintaining this same freedom for others, provided that the original source and author(s) are credited.Dongwoo Kim, Jae-Yong Chun, Kyeong-Yeoll Lee
