# Supplementary material for: Morphometric and molecular characterization of populations of Pratylenchus kumamotoensis and P. pseudocoffeae (Nematoda, Pratylenchidae) newly recorded in Korea
Source: Zookeys. 2016 Jun 22;(600):1–5. doi: 10.3897/zookeys.600.8508 (PMC4926677; doi:10.3897/zookeys.600.8508)
Supplement: Supplementary material 1 — Analysis of ITS2 and D2-d3 segment sequences of Pratylenchus kumamotoensis and Pratylenchus pseudocoffeae. [file zookeys-600-001-s001.doc]

**Supplementary data**

**Figure S1**

KT175515 CATGTGTGTGAGCCACAATGATGGTCAAACTTGTTTCGCAAGTTGAAAAGATCTATCCCC 60

LC030317 ............................................................ 60

LC030316 .....................................................--..... 60

LC030318 ............................................................ 60

LC030314 ............................................................ 60

LC030319 ...........A.............................C........T..--.A... 60

LC030313 ...........A.............................C........T..--.A... 60

LC030315 ...........A.............................C........T..--.A... 60

LC030312 .....T.....A.............................CG.......T..--.A... 60

***** *****.***************************** *******:** *.***

KT175515 ACAGATTATTGGGATTTGGAACTCGCTGTCACTGATTGGTGGCGTGAAGGCGCAACGGCT 120

LC030317 ............................................................ 120

LC030316 ............................................................ 118

LC030318 ............................................................ 120

LC030314 ............................................................ 120

LC030319 ......A.C............................A...................... 118

LC030313 ......A.C............................A...................... 118

LC030315 ......A.C............................A...................... 118

LC030312 ......A.C.....................G.A....A...................... 118

******:* *********************.*:****.**********************

KT175515 AACGTTGGTGTCTGTGTGTTGCTGAGCAGTCGTCTTCGTCCGTGGCTGTAATGAGGCAAC 180

LC030317 ............................................................ 180

LC030316 ............................................................ 178

LC030318 ............................................................ 180

LC030314 ............................................................ 180

LC030319 .................................................G.......... 178

LC030313 .................................................G.......... 178

LC030315 .................................................G.......... 178

LC030312 .................................................G.......... 178

*************************************************.**********

KT175515 ACGGTAGGACTACAGGCCGGAGTGGCATGTGGTTTAAGACTTGATGAGCCCCTAGATGTG 240

LC030317 ............................................................ 240

LC030316 ............................................................ 238

LC030318 ............................................................ 240

LC030314 ............................................................ 240

LC030319 .............GT............................................. 238

LC030313 .............GT........A.................................... 238

LC030315 .............GT............................................. 238

LC030312 .............GT............................................. 238

*************. ********.************************************

KT175515 GGACGCCAACACAACCCCTTTTCAAATACATTTTTTCAATATAAACAACAAATTCTAGTC 300

LC030317 ............................................................ 300

LC030316 ............................................................ 298

LC030318 ............................................................ 300

LC030314 ............................................................ 300

LC030319 ......................................................A..... 298

LC030313 .................................C....................A..... 298

LC030315 ......................................................A..... 298

LC030312 ............................................................ 298

********************************* ********************.*****

KT175515 TTATCGGTGGATCACTCGGCTCGTAGGTCGATGAAGAACGCAGCTAACTGCGATAAATAG 360

LC030317 ............................................................ 360

LC030316 ............................................................ 358

LC030318 ............................................................ 360

LC030314 ............................................................ 360

LC030319 ............................................................ 358

LC030313 ............................................................ 358

LC030315 ............................................................ 358

LC030312 ............................................................ 358

************************************************************

KT175515 TGTGAACTGCAGAAACATTGAGCACTAAAGATTCGAATGCACATTGCGCCATTGGAATAA 420

LC030317 ............................................................ 420

LC030316 ............................................................ 418

LC030318 ............................................................ 420

LC030314 ............................................................ 420

LC030319 ..............................C............................. 418

LC030313 ..............................C............................. 418

LC030315 ..............................C............................. 418

LC030312 ..............................C.........................G... 418

******************************.*************************.***

KT175515 TCATCCTTTGGCACGCCTGGTTCAGGGTCGCAAACCATAAACGCATCACAATTGCGTGAC 480

LC030317 ............................................................ 480

LC030316 ..........................-................................. 477

LC030318 ............................................................ 480

LC030314 ............................................................ 480

LC030319 ............................................................ 478

LC030313 ............................................................ 478

LC030315 ............................................................ 478

LC030312 ............................................................ 478

************************** *********************************

KT175515 TGCAAGTAAATATTCACATTGTGTATATTCAAATAGAAATTTCATGTGTTGTTTGCTGCA 540

LC030317 ................................G........................... 540

LC030316 .........................C.................................. 537

LC030318 ............................................................ 540

LC030314 ............................................................ 540

LC030319 G.....CG................................................A--- 538

LC030313 G.....CG................................................A--- 538

LC030315 G.....CG................................................A--- 538

LC030312 G......G.................................................--- 538

***** .***************** ******.***********************:

KT175515 TATATGCGGATGATTTTCAAAGTTGGTCTGTACATTTGAATTCATCATGAAGGTGTATTG 600

LC030317 ............................................................ 600

LC030316 ............................................................ 597

LC030318 ..................................................G......... 600

LC030314 ............................................................ 600

LC030319 ............................................................ 595

LC030313 ............................................................ 595

LC030315 ............................................................ 595

LC030312 ............................................................ 595

**************************************************.*********

KT175515 TGAGCTGTCACCTGCGCATTATATAGCAGCGCACATTTCGGCCAATGGCCAAATTCACAT 660

LC030317 ............................................................ 660

LC030316 ............................................................ 657

LC030318 ............................................................ 660

LC030314 ..................................................G......... 660

LC030319 --...................................C...................... 653

LC030313 --.......................................................... 653

LC030315 --.......................................................... 653

LC030312 --......................................................G... 653

*********************************** ************.*****.***

KT175515 TTTC 664

LC030317 .... 664

LC030316 .... 661

LC030318 .... 664

LC030314 .... 664

LC030319 .... 657

LC030313 .... 657

LC030315 .... 657

LC030312 .... 657

****

**Figure S2**

LC030338 CACACACCATGTATTGGTCAAACTTTGTTTTTTATGTGTGAGAGCACTCCTCATCAGATA 60

LC030337 ...........................................................G 60

LC030339 ............................................................ 60

FR691856 ............................................................ 60

FR692276 ............................................................ 60

KT175523 ............................................................ 60

KT971367 ............................................................ 60

***********************************************************.

LC030338 TGAGCGCGATGAGTTTTTGTATGTGAGTGCAAGTTGTATGTTGTACTCAGTTGTTGAGTG 120

LC030337 ............................................................ 120

LC030339 ............................................................ 120

FR691856 ............................................................ 120

FR692276 ............................................................ 120

KT175523 ............................................................ 120

KT971367 ............................................................ 120

************************************************************

LC030338 TGTGTCGTCACACATACAGCGTCTATCCGCTTAACCGCGCACATGTGAGAGCTGTGTGTG 180

LC030337 ............................................................ 180

LC030339 ............................................................ 180

FR691856 ............................................................ 180

FR692276 ......................................................C..... 180

KT175523 ............................................................ 180

KT971367 ............................................................ 180

****************************************************** *****

LC030338 TGTGTGCGCGGTTCCATCCGGTGTTTTTTGTGGATGGCTTTTGCTATGGAGCGGTCAAGA 240

LC030337 ............................................................ 240

LC030339 ............................................................ 240

FR691856 ............................................................ 240

FR692276 .........................................A.................. 240

KT175523 ............................................................ 240

KT971367 ............................................................ 240

*****************************************:******************

LC030338 AAACGGCTAACGCTGGTGTCTATGTGTTGCTGAGCAGTCGTCTTCGTCCGTGGCTGTGAT 300

LC030337 ............................................................ 300

LC030339 ............................................................ 300

FR691856 ............................................................ 300

FR692276 ............................................................ 300

KT175523 ............................................................ 300

KT971367 ............................................................ 300

************************************************************

LC030338 GAGGCAATGCGGTAGGGCCTGTATACGGTGTGTGTATGCATGGCTTAAGACTTGATGAGC 360

LC030337 ............................................................ 360

LC030339 ............................................................ 360

FR691856 ............................................................ 360

FR692276 ............................................................ 360

KT175523 ............................................................ 360

KT971367 ............................................................ 360

************************************************************

LC030338 CCATTTATTGTGTGTGGGACGCCAGCACCCCCTTTTTTCCAATATTTTTTTATGAAAAGT 420

LC030337 ............................................................ 420

LC030339 ............................................................ 420

FR691856 ........................................................G... 420

FR692276 ............................................................ 420

KT175523 ............................................................ 420

KT971367 ............................................................ 420

********************************************************.***

LC030338 GAAAAATTCTAGTCTTATCGGTGGATCACTCGGCTCGTGGGTCGATGAAGAACGCAGCTA 480

LC030337 ............................................................ 480

LC030339 ............................................................ 480

FR691856 ............................................................ 480

FR692276 ............................................................ 480

KT175523 ............................................................ 480

KT971367 ............................................................ 480

************************************************************

LC030338 ACTGCGATAAATAGTGTGAACTGCAGAAACCTTGAACACAAAAGCTTCGAATGCACATTG 540

LC030337 ............................................................ 540

LC030339 ............................................................ 540

FR691856 ............................................................ 540

FR692276 ........................G................................... 540

KT175523 ............................................................ 540

KT971367 ............................................................ 540

************************.***********************************

LC030338 CACCATGGGAGTCTTATCCCTTGGTACGCCTGGTTCAGGGTCGTAAACCCTAAAACGCAT 600

LC030337 ............................................................ 600

LC030339 ............................................................ 600

FR691856 ............................................................ 600

FR692276 ............................................................ 600

KT175523 ............................................................ 600

KT971367 ............................................................ 600

************************************************************

LC030338 ACAAATGCGTCTCTGTGATAGCATCACTTATATGGGCATCATCAAGTGTTACTATATAAT 660

LC030337 ............................................................ 660

LC030339 ............................................................ 660

FR691856 ............................................................ 660

FR692276 ............................................................ 660

KT175523 ............................................................ 660

KT971367 ............................................................ 660

************************************************************

LC030338 TCATGAAAAGTTGCACACAATTGGTGTGATGCGAGGTAGGGCACTCGACTTAGTAATTGG 720

LC030337 ............................................................ 720

LC030339 ............................................................ 720

FR691856 .................................................C.......... 720

FR692276 ............................................................ 720

KT175523 ............................................................ 720

KT971367 ............................................................ 720

************************************************* **********

LC030338 GATTACTAAACAATAGTCTAGTGCTCATCGGGGCAGACACCTCTCGAATTGCATCTGGCC 780

LC030337 ............................................................ 780

LC030339 ............................................................ 780

FR691856 ............................................................ 780

FR692276 ............................................................ 780

KT175523 ............................................................ 780

KT971367 ............................................................ 780

************************************************************

LC030338 GAATCACGTGTGTGTGCGTGTGTAACTTTTGAGTGTGTGTGTGTGTGTTTTTTTGCTCAC 840

LC030337 ............................................................ 840

LC030339 ............................................................ 840

FR691856 ............................................................ 840

FR692276 ............................................................ 840

KT175523 .....................................------................. 834

KT971367 ..........................................-----............. 835

************************************* *************

LC030338 TGTAATTGAATTTTC 855

LC030337 ............... 855

LC030339 ............... 855

FR691856 ............... 855

FR692276 ............... 855

KT175523 ............... 849

KT971367 ............... 850

***************

**Figure S3**

JX144360 GAGGGAAAGTTGCAAAGCACTTTGAAGAGAGAGTTAAAGAGGACGTGAAACCGATGAGAT 60

KT175528 ............................................................ 60

************************************************************

JX144360 GGAAACGGATAGAGTCAGCGTATCTGGCCTGTATTCATCCGCACTCCCATTGGCAGCAGG 120

KT175528 ..........................................G................. 120

******************************************.*****************

JX144360 GAGTTGCTCTCCAGATTGGGACTGATTTTTGCTTGCCCAGTGGTCGTGTGGTGCATTTGC 180

KT175528 ............................................................ 180

************************************************************

JX144360 AGGTGGAGTGCGTCGAGGCGCTGGGCACGGCTGCATGAACTCAGTTTTGAGGCCAGCTTG 240

KT175528 ............................................................ 240

************************************************************

JX144360 CTGGTACCCAAATTGGGGGATTGCTGTTCGTACTCAGTGTCTGCTGGCAAGGCATACGGG 300

KT175528 ............................................................ 300

************************************************************

JX144360 TTCGGTTGGGCACCGAGCTGGCAGTCGGAAGCGGTCACATGTGACACGTGCTGTGCGGTC 360

KT175528 ............................................................ 360

************************************************************

JX144360 AGTTCGGTCCTGGCCGAGCTCACTAGCCTGATCTCGGCGTAAAAGCTGGTCATCTATCCG 420

KT175528 ............................................................ 420

************************************************************

JX144360 ACCCGTCTTGAAACACGGACCAAGGAGTTTATCGTGTGCGCAAGTCATTGGGCATTGAAA 480

KT175528 ............................................................ 480

************************************************************

JX144360 ACCCAAAGGCGCAATGAAAGTGAATGTCTCCGCAAGGAGCAGACGTGCGATCCCGGGCAC 540

KT175528 ............................................................ 540

************************************************************

JX144360 TACGGTGCCTGGGCGCAGCATGGCCCCATCCCNATTGCTTGCAATGGGGTGGAGGAAGAG 600

KT175528 ................................G........................... 600

********************************.***************************

JX144360 CGTGCGCGGTGAGACCCGAAAGATGGTGAACTATTCCTGAGCAGGATGAAGCCAGAGGAA 660

KT175528 ............................................................ 660

************************************************************

JX144360 ACTCTGGTGGAAGTCCGAAGCGATTCTGACGTGCAAATCGATCGTCTGACTTGGGTATAG 720

KT175528 ............................................................ 720

************************************************************

JX144360 GGGCGAAAGACTAATCGAACCATCTAGTAGCTGGTTCCTTCC 762

KT175528 .......................................... 762

******************************************

**Figure S4**

AF170444 GCACTTTGAAGAGAGAGTTAAAGAGGACGTGAAACCGATGAGATGGAAACGGACAGAGCT 60

KT175531 ............................................................ 60

KT971360 ............................................................ 60

************************************************************

AF170444 AGCGTATCTGGCTTGCATTCAGCTTATGCGGTCGCTGCCGATGCGTCGCTGACCTCCAGA 120

KT175531 ............................................................ 120

KT971360 ..................................G............A............ 120

********************************** ************.************

AF170444 TTGGGGCTTTGACTAGTCGGTCGGTGGCTGTGTGGTGCATTTGCAAGTGGAGTGCGTCGA 180

KT175531 ............................................................ 180

KT971360 ............................................................ 180

************************************************************

AF170444 GGCACTTGGGATGGCGGAATGAACTTGGCTTTGAGGCCAGCTTGCTGGTACCCGGGCCAG 240

KT175531 ............................................................ 240

KT971360 ............................................................ 240

************************************************************

AF170444 GGGATTTCTGTTCGTTCTAGGTGTTTTACGGTGGAACAAGGCTCTGCGAGCCTGGTTGGG 300

KT175531 ...........................................T................ 300

KT971360 ............................................................ 300

******************************************* ****************

AF170444 TGCCGAGCTGGGTGTCGGTGGCGGTCGCATGCGACACGTACTGTGCCCACCAGTTCGGTC 360

KT175531 .............................................-.............. 359

KT971360 ............................................................ 359

********************************************* **************

AF170444 CTGGCCCGAGCTCACTCCCTGTTCAATCTCGGCGTAAAAGCTGGTCATCTTTCCGACCCG 420

KT175531 ....-....................................................... 418

KT971360 ............................................................ 418

**** *******************************************************

AF170444 TCTTGAAACACGGACCAAGGAGTTTATCGTGTGCGCGAGTCATTGGGCGTTGAAAACCCA 480

KT175531 ............................................................ 478

KT971360 ............................................................ 478

************************************************************

AF170444 AAGGCGCAATGAAAGTAAATGTATCCATCCGGAGCTGATGTGCGACCCTGGTCACTGCGG 540

KT175531 ............................................................ 538

KT971360 ............................................................ 538

************************************************************

AF170444 TGGCCAGGAGCAGCATGGCCCCATCCTGACTGCTTGCAGTGGGGTGGAGGAAGAGCGTAC 600

KT175531 ............................................................ 598

KT971360 ............................................................ 598

************************************************************

AF170444 GCGATGAGACCCGAAAGATGGTGAACTATTCCTGAGCAGGATGAAGCCAGAGGAAACTCT 660

KT175531 ............................................................ 658

KT971360 ............................................................ 658

************************************************************

AF170444 GGTGGAAGTCCGAAGCGATTCTGACGTGCAAATCGATCGTCTGACTTGGGTATAGGGGCG 720

KT175531 ............................................................ 718

KT971360 ............................................................ 718

************************************************************

AF170444 AAAGACTAATCGAACCATC 739

KT175531 ................... 737

KT971360 ................... 737

*******************

**Figure S5**

*P. pseudocoffeae* FR691856 (Iran)

*P. pseudocoffeae* FR692276 (Iran)

*P. pseudocoffeae* LC030338 (Japan)

*P. pseudocoffeae* LC030339 (Japan)

*P. pseudocoffeae* KT971367 (Costa Rica)

*P. pseudocoffeae* LC030337 (Japan)

***P. pseudocoffeae* KT175523 (Geumsan, Korea)**

*P. agilis* KC952982 (China)

*P. scribneri* JX046932 (China)

*P. alleni* jx081545 (Canada)

*P. gutierrezi* FJ712931 (Guatemala)

*P. loosi* JN091969 (Japan)

*P. jaehni* FJ712940 (Brazil)

*P. floridensis* GQ988375 (USA)

*P. parafloridensis* GQ988377 (USA)

*P. hippeastri* KJ001717 (Israel)

*P. coffeae* FJ712905 (Vietnam)

*P. speijeri* KF974739 (China)

*P. japonicus* KF452049 (Japan)

*P. zeae* KF765428 (China)

*P. bhattii* JN244272 (China)

*P. crenatus* FJ712915 (Finland)

*P. thornei* FJ713004 (Spain)

*P. mediterraneus* FJ712950 (Israel)

*P. neglectus* JX228136 (China)

*P. convallariae* HM469448 (China)

*P. pinguicaudatus* FJ712996 (Tunisia)

*P. brachyurus* JN020927 (China)

*P. fallax* FJ712920 (Belgium)

*P. lentis* AM933158 (Italy)

*P. vulnus* FR692320 (Italy)

*P. kumamotoensis* LC030315 (Kumamoto, Japan)

*P. kumamotoensis* LC030313 (Kagosima, Japan)

*P. kumamotoensis* LC030319 (Oita, Japan)

*P. kumamotoensis* LC030312 (Kagosima, Japan)

*P. kumamotoensis* LC030318 (Oita, Japan)

***P. kumamotoensis* KT175515 (Chilgok, Korea)**

*P. kumamotoensis* LC030314 (Kumamoto, Japan)

*P. kumamotoensis* LC030317 (Oita, Japan)

*P. kumamotoensis* LC030316 (Kumamoto, Japan)

*P. bolivianus* HM469446 (China)

*P. goodeyi* KM874803 (China)

100

100

56

100

100

43

16

43

47

70

100

91

100

100

100

100

72

52

84

86

100

97

91

47

47

34

7

21

54

64

99

77

94

80

100

62

100

0.05

**Figure S6**

*P. parafloridensis* GU214114 (USA)

*P. hippeastri* KJ001715 (Israel)

*P. floridensis* GU214117 (USA)

*P. araucensis* FJ463258 (Colombia)

*P. gutierrezi* AF170441 (Guatemala)

*P. scribneri* JX047002 (China)

*P. agilis* EU130841 (USA)

***P. pseudocoffeae* KT175531 (Geumsan, Korea)**

*P. pseudocoffeae* AF170444 (USA)

*P. pseudocoffeae* KT971360 (Costa Rica)

*P. loosi* JN091970 (Japan)

*P. speijeri* KF974713 (China)

*P. coffeae* KC490925 (China)

*P. japonicus* KF385445 (Japan)

*P. pratensis* AM231932 (Portugal)

*P. brachyurus* EU130842 (Japan)

*P. crenatus* KM580544 (Czech Republic)

*P. dunensis* AM230950 (England)

*P. penetrans* JX046990 (Netherland)

*P. kumamotoensis* JX144360 (Japan)

***P. kumamotoensis* KT175528 (Chilgok, Korea)**

*P. vulnus* JQ003994 (Japan)

*P. pseudopratensis* JX261965 (Iran)

*P. zeae* EU130894 (South Africa)

*P. delattrei* JX261948 (Iran)

*P. thornei* EU130880 (USA)

*P. neglectus* JX046969 (China)

*P. brzeskii* AM231928 (France)

100

100

99

100

99

58

100

68

64

83

81

28

33

95

99

100

100

100

69

37

61

100

100

57

100

0.02
